# HBV reactivation and its effect on survival in HBV-related hepatocarcinoma patients undergoing transarterial chemoembolization combined with tyrosine kinase inhibitors plus immune checkpoint inhibitors

**DOI:** 10.3389/fcimb.2023.1179689

**Published:** 2023-05-01

**Authors:** Jiaming Shen, Xia Wang, Ningning Wang, Shifei Wen, Guangde Yang, Li Li, Juanjuan Fu, Xiucheng Pan

**Affiliations:** Department of Infectious Disease, The Affiliated Hospital of Xuzhou Medical University, Xuzhou, China

**Keywords:** HBV-related HCC, TACE, TKIs, ICIS, HBV reactivation

## Abstract

**Objective:**

This study aimed to access hepatitis B virus (HBV) reactivation and its effect on survival in HBV-related hepatocarcinoma (HCC) patients who underwent transarterial chemoembolization (TACE) combined with tyrosine kinase inhibitors (TKIs) plus immune checkpoint inhibitors (ICIs).

**Methods:**

In this single-center retrospective study, we enrolled 119 HBV-related unresectable advanced HCC patients receiving TACE combined with TKIs plus ICIs. Risk factors for HBV reactivation were analyzed by logistic regression. Kaplan-Meier method was applied to draw the survival curve, and log-rank test was used to compare survival between patients with and without HBV reactivation.

**Results:**

A total of 12 patients (10.1%) encountered HBV reactivation in our study, of which only 4 patients received antiviral prophylaxis. The incidence of HBV reactivation was 1.8% (1/57) in patients with detectable baseline HBV DNA and 4.2% (4/95) in patients with antiviral prophylaxis respectively. Lack of prophylactic antiviral treatment (OR=0.047, 95%CI 0.008-0.273, *P*=0.001) and undetectable HBV DNA (OR=0.073, 95%CI 0.007-0.727, *P*=0.026) were independent risk factors for HBV reactivation. The median survival time (MST) for all patients was 22.4 months. No survival difference was observed in patients with or without HBV reactivation. (MST: undefined vs 22.4 months, log-rank test: *P*=0.614).

**Conclusion:**

HBV reactivation could occur in HBV-related HCC patients who treated with TACE in combination with TKIs plus ICIs. Before and during the combination treatment, it is necessary to routinely monitor HBV DNA and to take effective prophylactic antiviral therapy.

## Introduction

Globally, hepatocellular carcinoma (HCC) is the six most common cancer and the third leading cause of cancer-related death. Despite the progress made in early detection, most HCC patients are firstly diagnosed at an unresectable or advanced stage (Chan et al., 2016) in China, and have a poor prognosis. Due to loss of access to radical treatment, these patients require interventional therapy or/and systemic therapy. Transarterial chemoembolization (TACE), tyrosine kinase inhibitors (TKIs) and immune checkpoint inhibitors (ICIs) such as programmed cell death protein 1(PD-1) and its ligand (PD-1/L1) inhibitors are being widely used in patients with advanced or unresectable HCC (Gordan et al., 2020). TACE was routinely recommended to manage intermediate and advanced HCC, in fact, many studies have demonstrated that therapy of TACE in combination with TKI such as sorafinib, lenvatinib and other TKIs may have a synergistic anticancer activity and improve outcomes compared with TACE or a single drug for advanced HCC (Choi et al., 2013; Zhu et al., 2014; Fu et al., 2021). Recently, combination of atezolizumab (an anti-PD-L1 antibody) and bevacizumab (VEGF inhibitor) has been recommended as first-line therapy for advanced HCC patients (Gordan et al., 2020), due to its significant improvement of overall survival (OS) and progression-free survival (PFS). Moreover, some studies reported that TACE in combination with TKIs plus ICIs significantly improve the clinical outcomes in advanced HCC patients compared with dual therapy or TACE monotherapy (Ju et al., 2021; Cai et al., 2022; Zhu et al., 2023).

Hepatitis B virus (HBV) infection is a leading risk factor for HCC, especially in most Asian countries and regions (Chan et al., 2016). High baseline HBV DNA levels, immunosuppressive drugs and anti-cancer treatment can activate viral replication. The risk of HBV reactivation is proportional to the degree of immunosuppression and the intensity of treatment (Jang, 2014; Loomba and Liang, 2017). The definition of HBV reactivation varies from different guidelines, mainly including a sudden increase in viral load or reappearance of hepatitis B surface antigen (HBsAg) (Terrault et al., 2018; Lau et al., 2021). HBV reactivation could result in a variety of clinical events, ranging from mild hepatitis to fatal liver failure, sometimes even death (Jang, 2014). Therefore, it is necessary to screen patients with HBV-related HCC for HBV DNA, hepatitis B markers before and during anti-cancer treatment.

HBV reactivation can occur during and after treatment with TACE, TKIs and ICIs in patients with HBV-related HCC (Jang, 2014; Loomba and Liang, 2017). In recent years, a retrospective study by Zhang et al. firstly reported the rate and risk factor of HBV reactivation in HBsAg-positive cancer patients who receiving anti-PD-1/L1 monotherapy (Zhang et al., 2019). HBV reactivation was also observed to occur in patients treated with TKIs in combination with ICIs, and combination therapy was an independent risk factor for viral reactivation (Lei et al., 2023; Pan et al., 2022). To our best knowledge, previous clinical trials limited to patients with monotherapy and baseline undetectable HBV DNA or less than 100 IU/mL (Cheng et al., 2020; Ding et al., 2022). In real world, TACE combined with TKIs plus ICIs are usually used in unresectable advanced stage of HCC patients. However, there is no literature on HBV reactivation and its effect on survival of patients under this combined anticancer therapy. Here we conducted a retrospective study to access HBV reactivation and prognosis in HBV-related HCC patients receiving TACE combined with TKIs plus ICIs.

## Materials and methods

### Patients and study design

This was a retrospective study performed to review HBV-related HCC patients. Consecutive patients treated with combination therapy of TACE with TKI agents plus ICIs were collected from January 2020 to 31 December 2021 at the Affiliated Hospital of Xuzhou Medical University in China. The inclusion criteria were as follows: 1) aged between 18 and 85 years old; 2) diagnosed with HCC according to the American Association for the Study of Liver Diseases (AASLD) 2018 guidelines (Heimbach et al., 2018); 3) chronic or past HBV infection>6 months (HBsAg positive/negative and HBcAb (hepatitis B core antibody) positive); 4) combined TACE and TKIs within 2 weeks before and after the first ICI treatment; 5) received TACE (at least one cycle) in combination with TKIs plus ICIs (at least one dose); 6) Child-Pugh grade A/B (B7). Exclusion criteria included co-infection with other virus infections, such as hepatitis C virus (HCV) and human immunodeficiency virus (HIV); survival time less than 3 months; or lack of regular liver function, serological markers, HBV DNA detection and imaging data during immunotherapy. This study was approved by the Ethics Committee of the Affiliated Hospital of Xuzhou Medical University and the informed consent was not required because of its retrospective nature. Eventually, a total of 119 patients were included in this study.

### Clinical and laboratory variables

Patient demographic characteristics and treatment history were obtained from the electronic medical record system of the Affiliated Hospital of Xuzhou Medical University. Data related to routine blood, blood biochemistry, alpha-fetoprotein (AFP), HBV DNA, HBV serum infection markers and imaging were collected before and during anticancer treatment.

### Outcome assessments

The primary study endpoint was the occurrence of HBV reactivation, which was defined as one of the following according to Asian-Pacific Association for the study of the liver (APASL) clinical practice guideline on HBV reactivation (Lau et al., 2021): for patients with chronic HBV infection (HBsAg-positive), 1) ≥2 log increase in HBV DNA levels from baseline levels; 2) >100 IU/ml in a person with undetectable HBV DNA at baseline; for patients with resolved HBV infection (HBsAg negative and anti-HBc positive), 1) Reverse HBsAg seroconversion, HBsAg-negative becomes HBsAg-positive; 2) HBV DNA-undetectable becomes HBV DNA-detectable.

Other endpoints of this study include death from all causes, loss to follow-up, treatment interruption, end of last treatment, 31 March 2022.

### Statistical analysis

Qualitative variables were described as the frequency (percentage), and the quantitative variables were described as the median (range). We analyzed categorical variables by χ^2^ or Fisher exact test, while continuous variables were compared by T test or Mann-Whitney U test, as appropriate. Univariate and multivariate logistic regression analyses were used to identity the risk factors of HBV reactivation. Kaplan-Meier method was applied to draw the survival curve, and log-rank test was used for survival analysis. All statistical tests, a *P* value <0.05 was considered statistically significant. We used GraphPad prism (version 9.0) to generate the required pictures. Statistical analyses were performed using the SPSS statistical software (version 26.0).

## Results

### Patients

**Figure 1:**
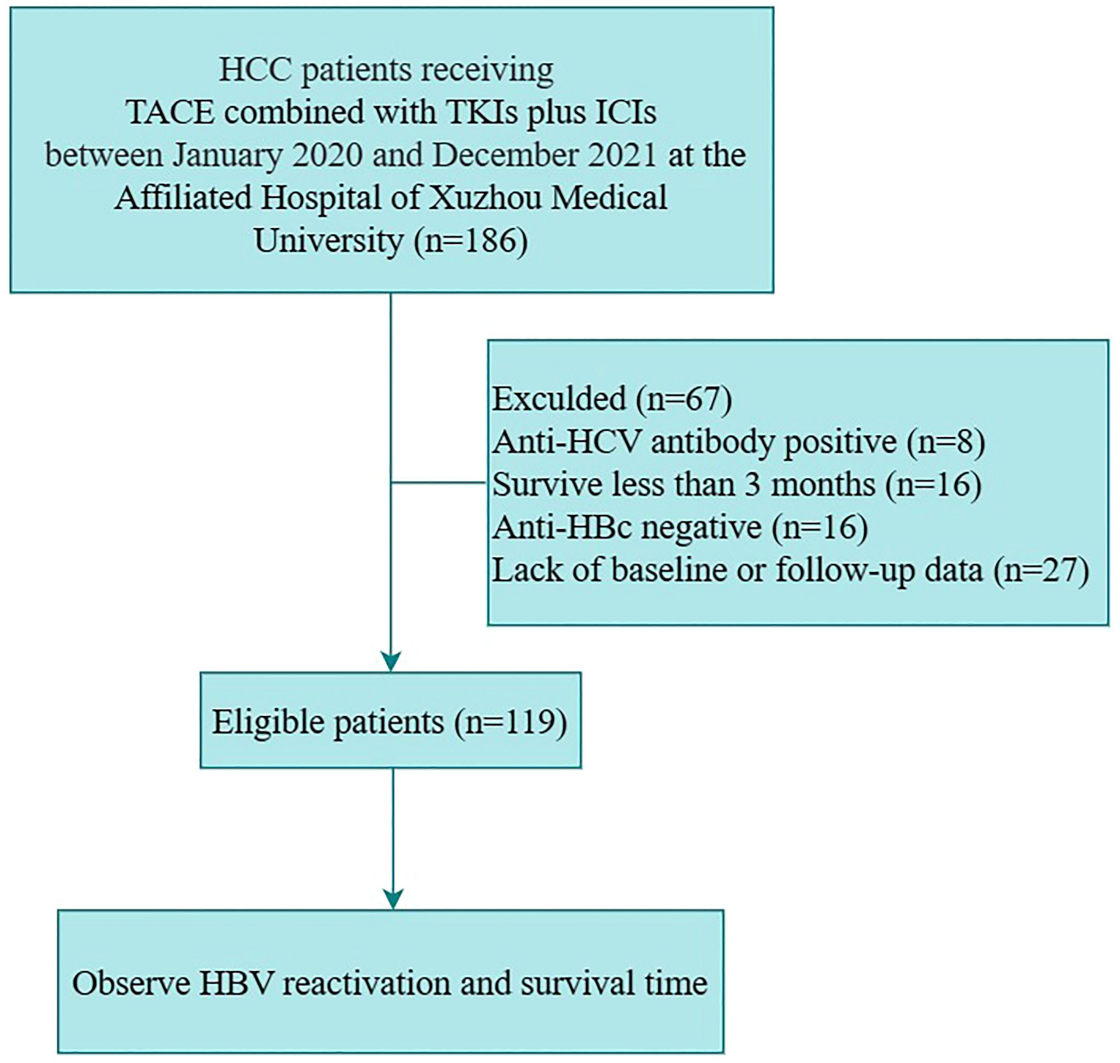
The patients flowchart. (Anti-HBc, antibody to hepatitis B core antigen; HBV, hepatitis B virus; HCC, hepatocarcinoma; HCV, hepatitis C virus; ICIs, immune checkpoint inhibitors; TACE, transarterial chemoembolization; TKIs, tyrosine kinase inhibitors).

Among 186 HCC patients treated with TACE in combination with TKIs plus ICIs between January 2020 to December 2021, 67 patients were excluded: 8 had co-infection with HCV, 16 were survived for less than 3 months, 16 were negative for hepatitis B core antibody, and 27 were lack of baseline or follow-up data. Ultimately, 119 patients were eligible for enrollment (**Figure 1**).

Baseline detail characteristics of enrolled patients are summarized in the **Table 1**. The ICIs drugs sintilimab (Innovent Biologics), camrelizumab (Hengrui Medicine) or tislelizumab (BeiGene) were given at a fixed dose of 200 mg every 3 weeks. The oral TKIs agents contained sorafenib (400 mg twice daily, Bayer), lenvatinib (8~12 mg/day, Eisai) and apatinib (250 mg/day, Hengrui Medicine). Briefly, these patients ranged in age from 19 to 82 years (median 57 years), with a male (n=109, 91.6%) predominance. At baseline, 113 patients (95.0%) were HBsAg positive, 57 patients (47.9%) had detectable HBV DNA levels (median 2170 IU/mL), and 30 patients had >2000 IU/mL of serum HBVDNA levels. A total of 95 patients received prophylactic antiviral therapy before receiving ICIs, the antiviral drugs were entecavir (ETV, 0.5 mg/day, Bristol-Myers Squibb or Chia Tai Qing), tenofovir disoproxil fumarate (TDF, 300 mg/day, GlaxoSmithKline K or Chia Tai Qing), tenofovir alafenamide fumarate (TAF, 25 mg/day, Gilead). Among the enrolled population, there were 104 cases (87.4%) of Child-Pugh grade A and 15 cases (12.6%) of grade B. According to the Barcelona Clinic Liver Cancer (BCLC) staging system, all patients were middle-advanced HCC patients, with 75 cases (63.0%) and 44 cases (37.0%) of stage B and C, respectively.

**Table 1:** Baseline characteristics of enrolled patients.

Characteristics	Total (n=119)	HBV reactivation (n=12)	Non-reactivation (n=107)	*P* value
Age, years ≥60 <60	57(19-82) 46(38.7%) 73(61.3%)	53.5(19-72) 5(41.7%) 7(58.3%)	57(24-82) 41(38.3%) 66(61.7%)	0.455^a^ 1.000^b^
Gender Male Female	109(91.6%) 10(8.4%)	11(91.7%) 1(8.3%)	98(91.6%) 9(8.4%)	1.000^b^
History of alcoholism Yes No	30(25.2%) 89(74.8%)	5(41.7%) 7(58.3%)	25(23.4%) 82(76.6%)	0.301^b^
Antiviral prophylaxis Yes No	95(79.8%) 24(20.2%)	4(33.3%) 8(66.7%)	91(85.0%) 16(15.0%)	<0.001^b^
HBsAg Seropositive Seronegative	113(95.0%) 6(5.0%)	10(83.3%) 2(16.7%)	103(96.3%) 4(3.7%)	0.112^c^
HBeAg Seropositive Seronegative	15(12.6%) 104(87.4%)	2(16.7%) 10(83.3%)	13(12.1%) 94(87.9%)	1.000^b^
Child-pugh A B	104(87.4%) 15(12.6%)	10(83.3%) 2(16.7%)	94(87.9%) 13(12.1%)	1.000^b^
BCLC B C	75(63.0%) 44(37.0%)	8(66.7%) 4(33.3%)	67(62.6%) 40(37.4%)	1.000^b^
HBV DNA, IU/mL				
Undetectable Detectable >2000 ≤2000 Median(range)	62(52.1%) 57(47.9%) 30(25.2%) 89(74.8%) 0 (0-3.89×10^6^)	11(91.7%) 1(8.3%) 0(0%) 12(100%) 0 (0-173)	51(47.7%) 56(52.3%) 30(28.0%) 77(72.0%) 33 (0-3.89×10^6^)	0.004^b^ 0.077^b^ 0.004^d^
ALT, U/L	34 (8-123)	38.5 (14-110)	34 (8-123)	0.534^d^
ALT>1 ULN ALT ≤ 1 ULN ALT>2 ULN ALT ≤ 2 ULN	37(31.1%) 82(68.9%) 6(5.0%) 113(95.0%)	4(33.3%) 8(66.7%) 1(8.3%) 11(91.7%)	33(30.8%) 74(69.2%) 5(4.7%) 102(95.3%)	1.000^b^ 0.479^c^
AST, U/L	41 (15-225)	54 (17-113)	40 (15-225)	0.691^d^
TBil, μmol/L	16.8 (2.8-78.2)	16.35 (9.7-54)	17.1 (2.8-78.2)	0.678^d^
ALB, g/L	39.4 (25.1-48.9)	40.85 (31.4-48.1)	39.3 (25.1-48.9)	0.317^a^
AFP, ng/mL AFP≥200 AFP<200	200.9 (1-164935) 60(50.4%) 59(49.6%)	314.165 (1.75-21685) 7(58.3%) 5(41.7%)	180.7 (1-164935) 53(49.5%) 54(50.5%)	0.737^d^ 0.563^b^
WBC, ×10^9^/L	5 (1.4-32)	5.5 (2.8-10.1)	5 (1.4-32)	0.665^d^
NLR >2.5 ≤2.5	77(64.7%) 42(35.3%)	5(41.7%) 7(58.3)	72(67.3%) 35(32.7%)	0.149^b^
PLT, ×10^9^/L	115 (19-493)	131.5 (22-493)	115 (19-345)	0.336^d^

AFP, alpha fetoprotein; ALB, albumin; ALT, alanine aminotransferase; AST, aspartate aminotransferase; BCLC, Barcelona Clinic Liver Cancer; HBeAg, hepatitis B e antigen; HBsAg, hepatitis B surface antigen; HBV, hepatitis B virus; NLR, neutrophil to lymphocyte ratio; TBil, total bilirubin; ULN, upper limit of normal; WBC, leukocyte; PLT, platelet.

a: T test.

b: χ^2^ test.

c: Fisher exact test.

d: Mann-Whitney U test.

### HBV reactivation

Among the 119 enrolled people, a total of 12 (10.1%) patients experienced HBV reactivation with a median reactivation time of 5.7 months (range 1.9-17.5 months). Besides, six of the enrolled patients developed a certain degree of increase in viral load, but did not meet the reactivation criteria. Details of the 12 patients with HBV reactivation are shown in **Table 2**. 11 of 12 patients were male and had undetectable HBV DNA at baseline. At onset of HBV reactivation, the median HBV DNA level was 596 IU/mL (range, 31-38800 IU/mL). Of the patients with viral reactivation, only 4 of 12 patients received antiviral prophylaxis, 7 people started taking antiviral agents after immunotherapy or after HBV reactivation, the remaining 1 patient did not take anti-HBV drugs orally.

**Table 2:** Details of 12 patients with HBV reactivation.

Baseline						At reactivation				
NO.	Age (years)	Gender	HBV DNA IU/ml	HBsAg	Antiviral prophylaxis	Intervals (months)	TACE (frequency)	HBV DNA IU/ml	HBsAg	Antiviral treatment
1	35	M	Undetectable	+	ETV	17.5	6	2130	+	ETV
2	50	M	Undetectable	+	/	15.0	6	688	+	ETV
3	63	M	Undetectable	+	/	2.8	3	107	+	ETV
4	49	M	Undetectable	+	ETV	4.2	3	3160	+	TAF
5	71	M	Undetectable	+	/	12.0	7	553	+	ETV
6	47	M	Undetectable	+	/	9.9	8	512	+	ETV
7	54	M	Undetectable	+	/	8.1	4	1240	+	ETV
8	72	M	Undetectable	–	/	2.5	2	31	–	ETV
9	66	M	Undetectable	–	/	1.9	2	113	–	ETV
10	53	M	Undetectable	+	/	2.6	2	639	+	/
11	19	F	Undetectable	+	ETV	6.9	2	153	+	ETV
12	64	M	173	+	ETV	4.4	1	38800	+	ETV

ETV, entecavir; F, female; HBsAg, hepatitis B surface antigen; M, male; NO., number; TACE, transarterial chemoembolization; TAF, tenofovir alafenamide fumarate.

In all patients with positive HBsAg, 8.8% (10/113) experienced viral reactivation. The rates of HBV reactivation were 1.8% (1/57) and 4.2% (4/95) in patients with detectable baseline HBV DNA and antiviral prophylaxis, respectively. It is worth noting that HBV reactivation occurred in 2 patients with HBsAg negativity (See **Supplementary Table 1**). At onset of HBV reactivation, their HBV DNA became detectable and HBsAg remained negative.

### HBsAg seroclearance

Among 113 HBsAg-positive patients, we observed that 3 patients achieved HBsAg seroclearance (**Table 3**). 2 patients did not receive prophylactic antiviral treatment, but initiated oral nucleoside/nucleotide analogs (NAs) therapy during immunotherapy. At the time of HBsAg seroclearance, all 3 patients took ETV orally and had undetectable HBV DNA.

**Table 3:** Clinical characteristics of 3 patient who achieved HBsAg seroclearance.

At baseline			
Age, years	59	51	49
Gender	M	M	F
Antiviral prophylaxis	No	Yes, ETV	No
HBV DNA	undetectable	undetectable	3160 IU/ml
HBsAg	(+)	(+)	(+)
ALT, U/L	17	62	51
TBil, μmol/L	12.5	24.5	16.4
ALB, g/L	30.8	39.1	33.1
Child-pugh	A	A	B
BCLC	C	B	B
At time of HBsAg seroclearance			
Antiviral treatment	Yes, ETV	Yes, ETV	Yes, ETV
HBV DNA	undetectable	undetectable	undetectable
Anti-HBs	(-)	NA	NA
Interval, months	8.4	6.6	15

AFP, alpha fetoprotein; ALB, albumin; ALT, alanine aminotransferase; Anti-HBc, antibody to hepatitis B core antigen; BCLC, Barcelona Clinic Liver Cancer; ETV, entecavir; F, female; HBsAg, hepatitis B surface antigen; NA, not available; TBil, total bilirubin.

### Factors associated with HBV reactivation

Results of univariate and multivariate logistic regression analysis for HBV reactivation are displayed in **Table 4**. In univariate analysis, undetectable HBV DNA at baseline and without antiviral prophylaxis were considered as potential risks for HBV reactivation with *P*<0.05. In multivariate analysis, lack of prophylactic antiviral treatment (OR=0.047, 95%CI 0.008-0.273, *P*=0.001) and undetectable HBV DNA (OR=0.073, 95%CI 0.007-0.727, *P*=0.026) remained independent risk factors for HBV reactivation. Patients with HBsAg positive at baseline (OR=2.144, 95%CI 0.217-21.210, *P*=0.514) or NLR ≤2.5 (OR=0.215, 95%CI 0.044-1.053, *P*=0.058) were at risk for viral reactivation, although not statistically significant.

**Table 4:** Univariate and multivariate logistic regression analysis for risk factors of HBV reactivation.

	Univariate		Multivariate	
	OR(95%CI)	*P*	OR(95%CI)	*P*
Age (≥60years)	1.150 (0.342-3.864)	.821		
Gender (male)	1.010 (0.117-8.742)	.993		
History of alcoholism (yes)	2.343 (0.684-8.031)	.176		
Antiviral prophylaxis (yes)	0.088 (0.024-0.327)	<.001	0.047 (0.008-0.273)	.001
HBsAg (+)	0.194 (0.032-1.195)	.077	2.144 (0.217-21.210)	.514
HBeAg (+)	1.446 (0.285-7.345)	.656		
HBV DNA (detectable)	0.083 (0.010-0.664)	.019	0.073 (0.007-0.727)	.026
ALT, U/L	1.007 (0.985-1.029)	.538		
ALT >1 ULN	1.121 (0.315-3.986)	.860		
TBil	1.015 (0.969-1.063)	.523		
ALB	1.067 (0.942-1.209)	.308		
Child-pugh (B)	1.446 (0.285-7.346)	.656		
BCLC (C)	0.838 (0.237-2.960)	.783		
AFP (≥200 ng/ml)	1.426 (0.426-4.777)	.565		
WBC	0.972 (0.806-1.171)	.764		
NLR (>2.5)	0.347 (0.103-1.172)	.088	0.215 (0.044-1.053)	.058
PLT	1.005 (0.999-1.012)	.113		

AFP, alpha fetoprotein; ALB, albumin; ALT, alanine aminotransferase; BCLC, Barcelona Clinic Liver Cancer; CI, confidence intervals; HBeAg, hepatitis B e antigen; HBsAg, hepatitis B surface antigen; NLR, neutrophil to lymphocyte ratio; OR, odds ratio; TBil, total bilirubin; WBC, leukocyte; ULN, upper limit of normal; PLT, platelet.

### Survival time

**Figure 2:**
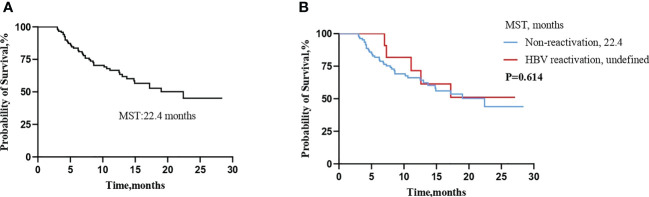
Kaplan-Meier curve for over survival (OS) time **(A)** in all enrolled patients, **(B)** in patients with or without HBV reactivation. (MST, median survival time).

A total of 45 (37.8%) patients died during the observation period in this study. The median survival time (MST) for all patients was 22.4 months (**Figure 2A**). There was no survival difference in patients with or without HBV reactivation. (MST:22.4 months vs undefined, log-rank test: *P*=0.614) (**Figure 2B**).

## Discussion

This is a real-world study that retrospectively analyzed the clinical data of patients with HBV-related HCC treated by TACE in combination with TKIs plus ICIs. This is also the first analysis of the rates of HBV reactivation in unresectable HCC patients undergoing this combination therapy. Data from our research showed that 10.1% patients encountered HBV reactivation. No significant survival difference was observed between patients with and without HBV reactivation. Lack of prophylactic antiviral treatment was an independent risk factor for HBV reactivation.

Systemic therapies, such as ICIs and TKIs, are recommended for patients with advanced HCC, as well as those with intermediate-stage HCC who are not candidates for local therapies (Vogel et al., 2022). HBV reactivation had been reported to occur in HBV-related HCC patients with TACE or systemic therapies (Jang, 2014; Loomba and Liang, 2017). The subsequent clinical manifestation of HBV reactivation varies from a slight elevation of ALT to liver failure, even death. Moreover, interruption or delay of anti-cancer therapy due to liver dysfunction could also have negative effect on the prognosis of patients (Jang, 2014). Lei et al. revealed that HCC patients received TKI alone or TKI-ICI treatment who encountered HBV reactivation had a significantly shorter median OS than those who did not (Lei et al., 2023). Of note, Liu et al. indicated that there was no significant difference in OS between HCC patients who received local therapy with or without HBV reactivation (Liu et al., 2020), which was similar to our finding. The possible reason was that severe HBV-associated hepatitis due to HBV reactivation was rare in our study, and timely initiation of antiviral therapy after HBV reactivation would improve patient survival. Furthermore, a previous review reported that baseline high HBV DNA levels was associated with poor survival in HBV-related HCC patients under local and systemic therapy (Yu and Kim, 2014). Most patients with HBV reactivation in our study had undetectable levels of baseline HBV DNA, which may improve outcomes to some extent.

Our results showed a higher overall incidence of HBV reactivation (10.1%) compared with previous studies of immunotherapy. The most likely reason we considered for the discrepancy was that all enrolled patients were treated with combination therapy of TACE and TKIs plus ICIs. A large cohort study in Korea showed that the incidence of HBV reactivation in HCC patients with Immunotherapy alone was 0.38% (Yoo et al., 2022). He et al. reported that the rates of HBV reactivation in HCC patients treated with ICIs combined with or without HAIC were 7.14%(6/84) and 0.85%(1/118), respectively (He et al., 2021). A previous article summarized that the incidence of HBV reactivation in patients treated with TACE ranged from 15% to 35% (Jang, 2014), and there was a dose-risk relationship between the intensity of TACE treatment and viral reactivation (Jang et al., 2011). In patients with positive HBsAg, TACE is a high-risk factor for HBV reactivation (Loomba and Liang, 2017). In addition, it had been reported that the incidence of HBV reactivation in HCC patients undergoing TKIs (lenvatinib, sorafenib, etc.) combined with PD-1 inhibitors (sintilimab, camrelizumab, etc.) therapy is higher than that in patients with TKIs monotherapy (Lei et al., 2023). Accordingly, the triple therapy of TACE combined with TKIs plus ICIs might cause the higher rates of HBV reactivation in our study.

In our study, we observed a total of 12 patients with HBV reactivation. Of these 12 patients, 4 patients developed HBV reactivation even with prophylactic ETV antiviral therapy. The rates of HBV reactivation were 4.2% (4/95) and 33.3% (8/24) in patients with and without antiviral prophylaxis, respectively. Lack of prophylactic antiviral therapy was an independent risk factor for HBV reactivation, which was consistent with the results of other study (Zhang et al., 2019). Even with prophylactic anti-HBV therapy, HCC patients receiving anticancer treatment may still encounter HBV reactivation, which had also been reported in several previous studies (Lao et al., 2013; Zhang et al., 2019; Yoo et al., 2022). On one hand, the possible reason was that patients developed viral resistance due to complex histories of NAs medication (Tenney et al., 2009; Guo et al., 2018). On the other hand, limited by the nature of the real-world retrospective study, we could not obtain accurate information on medication use and adherence. It was possible that irregular oral administration of NAs reactivated HBV replication (Yoo et al., 2022). In general, under the treatment of TACE combined with TKIs plus ICIs, HBV reactivation mostly occurred in patients who did not take prophylactic antiviral. It is necessary to take regular and effective antiviral treatment throughout.

One of the virologic risk factors for HBV reactivation is high baseline viral load (Jang, 2014; Loomba and Liang, 2017). Interestingly, in our study, most patients with HBV reactivation had undetectable baseline viral load. Patients with detectable baseline HBV DNA levels had lower rates of HBV reactivation (1.8%,1/57) than those with undetectable baseline HBV DNA (17.7%,11/62). None of the patients with baseline HBV DNA >2000 IU/ml developed viral reactivation. Multivariate analysis results showed that patients with undetectable baseline viral load were more at risk for HBV reactivation. The most likely reason for this discrepancy was that: compared with those with detectable baseline HBV DNA, a higher proportion of patients with undetectable baseline HBV DNA did not receive prophylactic antiviral therapy (24.2%,15/62 vs 15.8%,9/57). Preemptive antiviral treatment is important in HBV-related HCC comprehensive therapy.

A recent review of 13 ICIs-related studies with 2561 patients showed that chronic hepatitis B (CHB) patients were at higher risk of HBV reactivation than those with past infection (1.0% vs 0%) (Ding et al., 2022). However, we found that 2 patients with past HBV infection developed HBV reactivation. The level of serum HBV DNA changed from undetectable to detectable at reactivation, rather than HBsAg serological conversion. A previous study reported HBV reactivation in a patient with HCC with past HBV infection who treated by nivolumab. At the time of HBV reactivation, the level of HBsAg changed from negative to positive (Wong et al., 2021). Given that HBsAg-negative patients can develop HBV reactivation in the setting of TACE or TKI agents therapy (Peng et al., 2012; Jang et al., 2015; Lee et al., 2022), we cannot entirely attribute these two cases of HBV reactivation to ICIs therapy alone.

Notably, seroclearance of HBsAg occurred in 3 enrolled patients, who had been treated with ETV antiviral therapy prior to receiving ICIs. An observational study in Hong Kong reported that only 1 of 397 (<1%) HBsAg-positive patients was observed to encounter HBsAg seroclearance (Wong et al., 2021). Although ICIs are recognized to enhance immunity and help clear viral infection, the incidence of HBsAg seroclearance is low in the real-world setting.

HBV reactivation remains a key issue during the anti-cancer treatment of patients with HBV-related HCC. As a HBV persistent reservoir and key obstacle to cure CHB patients (Nassal, 2015), covalently closed circular DNA (cccDNA) is also one of the virological risk factors for HBV reactivation (Jang, 2014). HBV establishes its genome as a cccDNA in the nucleus of infected hepatocytes. NAs such as ETV, TDF, etc. and interferon-α can effectively inhibit HBV replication, but there is no effective drug against HBV cccDNA to date, so it is difficult to eradicate it from infected cells (Xia and Guo, 2020). In theory, viral reactivation after HBV replication can lead to detectable viremia in sufficient time even if only one copy of cccDNA remains (Shi and Zheng, 2020).

There were some other limitations in this study. First, this study was a single-center retrospective study with a small sample size. Second, the time interval of HBV DNA or HBsAg screening was inconsistent within and among enrolled patients, and the delay of testing might result in unobserved endpoint events in some patients during the study period. So, the incidence of HBV reactivation may be underestimated. Third, types of antiviral drugs taken by patients were inconsistent, as well as the dose of chemotherapeutic agents required for TACE, which have the potential to influence our results. Given these limitations, multicenter prospective studies should be needed.

In summary, HBV reactivation could occur in HBV-related HCC patients who treated with TACE in combination with TKIs plus ICIs. Before and during the combination treatment, it is necessary to routinely monitor viral load and HBV-related serological indicators, and to take effective prophylactic antiviral therapy.

## Data availability statement

The original contributions presented in the study are included in the article/**Supplementary Material**. Further inquiries can be directed to the corresponding author.

## Ethics statement

The studies involving human participants were reviewed and approved by The ethics committee of Xuzhou Medical University Affiliated Hospital (Ethics number: xyfy2022-KL085-01). Written informed consent for participation was not required for this study in accordance with the national legislation and the institutional requirements.

## Author contributions

JS: Resources, data curation, investigation, methodology, drafted and edited the manuscript. XW: Resources, data curation and manuscript writing. NW, SW, GY: data curation, data analysis, reviewed and revised the manuscript. LL, JF: analysis supervise, reviewed and revised the manuscript. XP: Conceptualization, supervision, review and editing. All authors contributed to the article and approved the submitted version.
