# Supplementary material for: HBV reactivation and its effect on survival in HBV-related hepatocarcinoma patients undergoing transarterial chemoembolization combined with tyrosine kinase inhibitors plus immune checkpoint inhibitors
Source: Front Cell Infect Microbiol. 2023 May 1;13:1179689. doi: 10.3389/fcimb.2023.1179689 (PMC10183577; doi:10.3389/fcimb.2023.1179689)
Supplement: Supplementary file 1 [file Table_1.docx]

**Supplement Table 1** Data of 2 baseline HBsAg-negative patients with HBV reactivation

| Age, years | 72 | 66 |
| --- | --- | --- |
| Gender | M | M |
| Antiviral prophylaxis | No | No |
| History of alcoholism | Yes | Yes |
| Baseline HBV DNA | undetectable | undetectable |
| Baseline HBsAg | (-) | (-) |
| ALT, U/L | 16 | 50 |
| TBil, μmol/L | 16.3 | 33.6 |
| ALB, g/L | 41.1 | 37.3 |
| Child-pugh | A | A |
| BCLC | B | B |
| At onset of HBV reactivation |  |  |
| Antiviral treatment | Yes, ETV | Yes, ETV |
| HBV DNA, U/L | 31 | 113 |
| HBsAg | (-) | (-) |
| ALT, U/L | 1027 | 62 |

ALT, alanine aminotransferase; ALB, albumin; BCLC, Barcelona Clinic Liver Cancer; HBsAg, hepatitis B surface antigen; HBV, hepatitis B virus; M, male; TBil, total bilirubin.
